# Chiral optical tweezers for optically active particles in the T-matrix formalism

**DOI:** 10.1038/s41598-018-36434-9

**Published:** 2019-01-10

**Authors:** Francesco Patti, Rosalba Saija, Paolo Denti, Giovanni Pellegrini, Paolo Biagioni, Maria Antonia Iatì, Onofrio M. Maragò

**Affiliations:** 1 CNR-IPCF, Istituto per i Processi Chimico-Fisici, I-98158 Messina, Italy; 2 Dipartimento di Scienze Matematiche e Informatiche, Scienze Fisiche e Scienze della Terra, Università di Messina, I-98166 Messina, Italy; 3 Dipartimento di Fisica, Politecnico di Milano, I-20133 Milano, Italy

## Abstract

Modeling optical tweezers in the T-matrix formalism has been of key importance for accurate and efficient calculations of optical forces and their comparison with experiments. Here we extend this formalism to the modeling of chiral optomechanics and optical tweezers where chiral light is used for optical manipulation and trapping of optically active particles. We first use the Bohren decomposition to deal with the light scattering of chiral light on optically active particles. Thus, we show analytically that all the observables (cross sections, asymmetry parameters) are split into a helicity dependent and independent part and study a practical example of a complex resin particle with inner copper-coated stainless steel helices. Then, we apply this chiral T-matrix framework to optical tweezers where a tightly focused chiral field is used to trap an optically active spherical particle, calculate the chiral behaviour of optical trapping stiffnesses and their size scaling, and extend calculations to chiral nanowires and clusters of astrophysical interest. Such general light scattering framework opens perspectives for modeling optical forces on biological materials where optically active amino acids and carbohydrates are present.

## Introduction

A chiral object is affected by the lack of symmetry under reflection^1^. Both radiation and material objects may have this property. The two chiral versions of an object are referred to as *enantiomers*. A great number of organic molecules, such as proteins and sugars, are characterized by optical activity, which has caused the claim that “optical activity provides a peephole into the fabric of universe”^2^. Since optical activity is related to the property of a material to sustain the propagation of circularly polarized waves with a speed of propagation that depends on the handedness of their polarization, gyrotropic studies can yield a wealth of information on optically active materials. The electromagnetic radiation can also be chiral, especially when we refer to left (LCP) and right (RCP) circular polarization. Its degree of chirality is measured through the *optical chirality* ${\mathscr{C}}$:

1 $${\mathscr{C}}=\frac{{\varepsilon }_{0}}{2}\overrightarrow{E}\cdot \overrightarrow{\nabla }\times \overrightarrow{E}+\frac{1}{2{\mu }_{0}}\overrightarrow{B}\cdot \overrightarrow{\nabla }\times \overrightarrow{B}$$

introduced by Lipkin in the early sixties^3^. The main feature of the interaction between a chiral medium and a chiral radiation is that it depends on the respective handedness^4,5^.

Mechanical effects of light^6,7^ stem from conservation laws^8,9^. In particular, optical tweezers^7^, tools based on a tightly focused laser beam, can grab particles^9^, cells^10^, viruses^11^, nanostructures^12^ and have been recently awarded the Nobel prize in Physics^13^ 2018. Besides the transfer of linear momentum that yields optical forces^6,7^, circularly polarized light may transfer also spin angular momentum^14^ yielding light-induced rotations on absorbing^15–17^ or birefringent particles^18–20^. Recently, chirality-dependent optical forces on small particles have been considered theoretically for the all-optical separation of enantiomers^21–24^. On the other hand, the mechanical interaction between chiral light and supramolecular chiral particles at the mesoscale has been studied with optical tweezers^7^ investigating the optomechanics of cholesteric liquid crystals^25–32^ (CLC). Their chiral properties result from the combination of birefringence^32^ and a supramolecular multishell structure that yield a chiral band gap and radially directed Bragg reflections over a specific frequency range^25^. Left-handed CLC solid microparticles have been synthesized^25^, optically trapped, and chiral rotations observed for the corresponding LCP light^30,32^. Thus, while chiral reflection is the key to understand the chiral optomechanics in these systems, modeling these structures is quite complex because of their intrinsic morphological anisotropy and inhomogeneity^25^.

Here, we devise a framework to calculate the optomechanical interaction between optically active chiral particles of any size or complexity and a chiral radiation field. Within a T-matrix framework^8,9,33,34^, we first show analytically that cross sections, optical forces and torques separate into helicity dependent and independent parts. Then, we study some practical examples, a complex resin particle with inner copper helices, the chiral optical trapping of optically active spherical particles, of a chiral nanowire, and of a particle cluster with optical properties corresponding to the amino acids discovered in the Murchison meteorite^35^.

## Results

### Analytical formulation of chiral optomechanics

The scattering problem for optically active spheres has been originally studied by Bohren^4,36^. Recently, the approach was extended to deal with aggregates of optically active particles^37^. We consider an optically active particle with an average refractive index ${n}_{{\rm{p}}}=\sqrt{\varepsilon /\mu }$ immersed in a non-dispersive, homogeneous medium of refractive index *n*_m_. The staring point for a macroscopic description of optical activity are the Drude-Born-Fedorov (DBF) constitutive relations^4,5^:

2 $$\overrightarrow{D}=\varepsilon \overrightarrow{E}+\frac{\alpha \varepsilon }{k}\overrightarrow{\nabla }\times \overrightarrow{E},$$

3 $$\overrightarrow{B}=\mu \overrightarrow{H}+\frac{\beta \mu }{k}\overrightarrow{\nabla }\times \overrightarrow{H}\mathrm{.}$$

If time symmetry is imposed, *α* = *β*. The adimensional chirality parameter *β* is related to the chiral refractive indices, ${n}_{L}=\bar{n}\mathrm{/(1}-\beta \bar{n})$ and ${n}_{R}=\bar{n}\mathrm{/(1}+\beta \bar{n})$, for left and right circularly polarized waves, respectively, where $\bar{n}={n}_{{\rm{p}}}/{n}_{{\rm{m}}}$ is the average refractive index relative to the medium (see Supplementary Information):

4 $$\beta =\frac{1}{2}(\frac{1}{{n}_{R}}-\frac{1}{{n}_{L}})\mathrm{.}$$

Thus, the real part of *β* is related with the different speed of propagation and its imaginary part with the different absorption of RCP and LCP electromagnetic waves. In addition, if the real part of *β* is positive the material is a right-handed medium and the LCP radiation propagates with a slower phase velocity, and *viceversa*.

Note that for an isotropic chiral medium the BDF constitutive equations satisfy the time-reversal symmetry, the duality transformations, and their validity is supported by experiments^1,2^. Moreover, it has been recently shown that conservation of energy is preserved both globally and locally^38^. All these facts consolidate the physical significance of the BDF relations and give a strong argument for their use in practical calculations. Noteworthy, other formulations of the constitutive relations are also suitable to describe optical activity^39^, for isotropic reciprocal chiral media^40^, and for composite chiral media^41^. However, a distinction between different approaches is possible only for arbitrarily time-dependent (not simply harmonic) processes or for inhomogeneous media. The various constitutive relations are equivalent to each other for time-harmonic fields. Here we consistently use the BDF relations in the framework of electromagnetic scattering theory from isotropic optically active particles from which we find a Helmholtz-type equation:

5 $${\nabla }^{2}[\begin{array}{c}\overrightarrow{E}\\ \overrightarrow{H}\end{array}]+{{\mathscr{K}}}^{2}[\begin{array}{c}\overrightarrow{E}\\ \overrightarrow{H}\end{array}]=\mathrm{0,}$$

where ${\mathscr{K}}$ is the non-diagonal chiral matrix:

6 $${\mathscr{K}}=\frac{k}{1-{\beta }^{2}\mu \varepsilon }(\begin{array}{cc}\beta \mu \varepsilon  & i\mu \\ -\,i\varepsilon  & \beta \mu \varepsilon \end{array})$$

and *k* is the wavevector in vacuum. Thus, since ${\mathscr{K}}$ is non-diagonal, in a chiral medium the fields are coupled with each other during their propagation. Bohren has shown that any field in the chiral medium can be described as an overlap of the circularly polarized fields, ${\overrightarrow{Q}}_{L}$ and ${\overrightarrow{Q}}_{R}$ (*Bohren decomposition*)^4,36,37^:

7 $$\{\begin{array}{rcl}{\overrightarrow{E}}_{P} & = & {\overrightarrow{Q}}_{L}-\frac{i}{{n}_{{\rm{p}}}}{\overrightarrow{Q}}_{R}\\ {\overrightarrow{H}}_{P} & = & -\,i{n}_{{\rm{p}}}{\overrightarrow{Q}}_{L}+{\overrightarrow{Q}}_{R}\mathrm{.}\end{array}$$

We now expand these internal fields in magnetic and electric multipoles, ${\overrightarrow{J}}_{Llm}^{\mathrm{(1)}},\,{\overrightarrow{J}}_{Rlm}^{\mathrm{(1)}},\,{\overrightarrow{J}}_{Llm}^{\mathrm{(2)}},\,{\overrightarrow{J}}_{Rlm}^{\mathrm{(2)}}$, with parity indices *p* = 1, 2 respectively^8^. The numerical values of the superscripts are related to the parity operator, *i.e*., by reflecting a magnetic or electric vector, its sign changes or does not change, so that the eigenvalue is either −1 (for *p* = 1) or 1 (for *p* = 2). The natural optical activity can be considered as generated by an appropriate superposition of these vector fields, involving interference between electric and magnetic multipoles. In particular, we have that:

8 $$(\begin{array}{rcl}{\overrightarrow{Q}}_{L} & = & \sum _{lm}\,{C}_{Llm}[{\overrightarrow{J}}_{Llm}^{\mathrm{(1)}}+{\overrightarrow{J}}_{Llm}^{\mathrm{(2)}}]\\ {\overrightarrow{Q}}_{R} & = & \sum _{lm}\,{C}_{Rlm}[-\,{\overrightarrow{J}}_{Rlm}^{\mathrm{(1)}}+{\overrightarrow{J}}_{Rlm}^{\mathrm{(2)}}],\end{array}$$

with indices *l* = 0, 1, ... and *m* = −*l*, ..., 0, ..., *l* related to the angular momentum of the multipoles, and:

9 $${\overrightarrow{J}}_{Llm}^{\mathrm{(1)}}={j}_{l}({k}_{L}r){\overrightarrow{X}}_{lm}^{\mathrm{(1)}}(r),\,{\overrightarrow{J}}_{Rlm}^{\mathrm{(1)}}={j}_{l}({k}_{R}r){\overrightarrow{X}}_{lm}^{\mathrm{(1)}}(r),$$

10 $${\overrightarrow{J}}_{Llm}^{\mathrm{(2)}}=\frac{1}{{k}_{L}}\overrightarrow{\nabla }\times {\overrightarrow{J}}_{Llm}^{\mathrm{(1)}},\,{\overrightarrow{J}}_{Rlm}^{\mathrm{(2)}}=\frac{1}{{k}_{R}}\overrightarrow{\nabla }\times {\overrightarrow{J}}_{Rlm}^{\mathrm{(1)}},$$

where the chiral wavevectors are *k*_*L*_ = *n*_*L*_*k* and *k*_*R*_ = *n*_*R*_*k*. The expansion coefficients, *C*_*Llm*_ and *C*_*Rlm*_, represent the so-called *optical activity tensor* that describes completely all phenomena related with chirality. By exploiting this approach, imposing the boundary conditions, the full scattering problem by chiral particles can be solved in the T-matrix formalism^42^ (see Supplementary Information), where the incident and scattered fields are expanded^8,43^ in terms of spherical Bessel J-multipoles with expansion coefficients ${W}_{lm}^{p^{\prime} }$ for the incident fields and spherical Hankel H-multipoles with coefficients ${A}_{lm}^{(p)}$ for the scattered fields:

11 $${A}_{lm}^{(p)}={T}_{lml^{\prime} m^{\prime} }^{(pp^{\prime} )}{W}_{l^{\prime} m^{\prime} }^{p^{\prime} }\mathrm{.}$$

Here ${T}_{lml^{\prime} m^{\prime} }^{(pp^{\prime} )}$ represent the elements of the T-matrix for the chiral particle. For the specific case of a chiral sphere, the T-matrix has a simple expression^37^ written in terms of coefficients, ${R}_{l}^{(pp^{\prime} )}$, in close analogy with the Mie coefficients, which are combinations of Riccati-Bessel and Riccati-Hankel functions dependent on the sphere size parameter and on the chiral refractive indices (see Supplementary Information):

12 $${T}_{lml^{\prime} m^{\prime} }^{(pp^{\prime} )}=-\,{R}_{l}^{(pp^{\prime} )}{\delta }_{ll^{\prime} }{\delta }_{mm^{\prime} }\mathrm{.}$$

In contrast to the Mie case, as expected, the T-matrix here is non-diagonal with respect to the parity index, *p*, since parity in not conserved for a chiral sphere, but the matrix is still symmetric, *i.e*., ${R}_{l}^{(pp^{\prime} )}={R}_{l}^{(p^{\prime} p)}$.

We can now exploit this approach to calculate the chiral optical forces generated in the chiral material-chiral radiation optomechanical interaction. First, we focus on a plane wave illumination, studying the simple example of a chiral sphere. Then we extend the approach to the case of optical tweezers.

### Plane wave illumination

For a chiral plane wave illumination we can write a simple expression for the radiation pressure force^9,44,45^ along the propagation direction, ${\hat{k}}_{0}$, in terms of the incident intensity, *I*_0_, the extinction and scattering cross sections, ${\tilde{\sigma }}_{ext}$ and ${\tilde{\sigma }}_{scat}$, and the anisotropy parameter, ${\tilde{g}}_{i}$:

13 $${\overrightarrow{F}}_{rad}=\frac{{n}_{{\rm{m}}}}{c}{I}_{0}[{\tilde{\sigma }}_{ext}-{\tilde{\sigma }}_{scat}{\tilde{g}}_{i}]\,{\hat{k}}_{0}=\frac{n}{c}{I}_{0}{\tilde{\sigma }}_{rad}{\hat{k}}_{0}\mathrm{.}$$

The general expressions for the cross sections and anisotropy parameter are then expressed in terms of the chiral matrix elements, ${R}_{l}^{(pp^{\prime} )}$:

14 $${\tilde{\sigma }}_{ext}^{\eta }=\frac{2\pi }{{k}_{{\rm{m}}}^{2}}\sum _{lpp^{\prime} }\,\mathrm{(2}l+\mathrm{1)}{R}_{l}^{(pp^{\prime} )}\,[{\delta }_{pp^{\prime} }-{(-1)}^{\eta }\,\mathrm{(1}-{\delta }_{pp^{\prime} })]$$

15 $${\mathop{\sigma }\limits^{ \sim }}_{scat}^{\eta }=\frac{2\pi }{{k}_{{\rm{m}}}^{2}}\,\sum _{lp}\,\sum _{p{\rm{^{\prime} }}p{\rm{^{\prime} }}{\rm{^{\prime} }}}\,(2l+1){R}_{l}^{(pp{\rm{^{\prime} }})\ast }{R}_{l}^{(pp{\rm{^{\prime} }}{\rm{^{\prime} }})}[{\delta }_{p{\rm{^{\prime} }}p{\rm{^{\prime} }}{\rm{^{\prime} }}}-{(-1)}^{\eta }\,(1-{\delta }_{p{\rm{^{\prime} }}p{\rm{^{\prime} }}{\rm{^{\prime} }}})]$$

16 $$\begin{array}{lll}{\mathop{\sigma }\limits^{ \sim }}_{scat}^{\eta }{{\mathop{g}\limits^{ \sim }}_{i}}^{\eta } & = & \frac{2\pi }{{k}_{{\rm{m}}}^{2}}\sum _{lp}\,{\rm{R}}{\rm{e}}\{\frac{l(l+2)}{l+1}\sum _{p{\rm{^{\prime} }}p{\rm{^{\prime} }}{\rm{^{\prime} }}}\,{R}_{l}^{(pp{\rm{^{\prime} }})}{R}_{l+1}^{(pp{\rm{^{\prime} }}{\rm{^{\prime} }})\ast }[{\delta }_{p{\rm{^{\prime} }}p{\rm{^{\prime} }}{\rm{^{\prime} }}}-{(-1)}^{\eta }\,(1-{\delta }_{p{\rm{^{\prime} }}p{\rm{^{\prime} }}{\rm{^{\prime} }}})]\\  &  & +\,\frac{2l+1}{l(l+1)}\sum _{p{\rm{^{\prime} }}{\rm{^{\prime} }}p{\rm{^{\prime} }}{\rm{^{\prime} }}{\rm{^{\prime} }}}\,{R}_{l}^{(pp{\rm{^{\prime} }}{\rm{^{\prime} }}{\rm{^{\prime} }})}{R}_{l+1}^{(p{\rm{^{\prime} }}p{\rm{^{\prime} }}{\rm{^{\prime} }}{\rm{^{\prime} }})\ast }[1-{\delta }_{pp{\rm{^{\prime} }}}][{\delta }_{p{\rm{^{\prime} }}{\rm{^{\prime} }}p{\rm{^{\prime} }}{\rm{^{\prime} }}{\rm{^{\prime} }}}-{(-1)}^{\eta }(1-{\delta }_{p{\rm{^{\prime} }}p{\rm{^{\prime} }}{\rm{^{\prime} }}})]\},\end{array}$$

where *δ* is the Kronecker delta, *k*_m_ = *n*_m_*k* is the wavevector in the surrounding medium, and *η* represents a polarization index, with *η* = 1 for LCP, *i.e*., a polarization unit vector ${\hat{c}}_{1}=(\hat{x}+i\hat{y})/\sqrt{2}$, or *η* = 2 for RCP, *i.e*., a polarization unit vector ${\hat{c}}_{2}=(\hat{x}-i\hat{y})/\sqrt{2}$. Thus, it is possible to show that all observables (cross sections, optical forces) are separated into a helicity independent part, corresponding to the value for linear polarization, and a second part that is summed or subtracted depending on the light helicity (see Supplementary Information). As an example, the expression for the extinction cross section for an optically active sphere is ${\tilde{\sigma }}_{ext}^{\mathrm{1,2}}={\tilde{\sigma }}_{ext}^{0}\pm {\tilde{\sigma }}_{ext}^{h}$, with:

17 $${\tilde{\sigma }}_{ext}^{0}=\frac{2\pi }{{k}_{{\rm{m}}}^{2}}\sum _{l}\,\mathrm{(2}l+\mathrm{1)}{Re}\{{R}_{l}^{\mathrm{(11)}}+{R}_{l}^{\mathrm{(22)}}\},\,{\tilde{\sigma }}_{ext}^{h}=\frac{2\pi }{{k}_{{\rm{m}}}^{2}}\sum _{l}\,\mathrm{2(2}l+\mathrm{1)}{Re}\{{R}_{l}^{\mathrm{(12)}}\mathrm{\}.}$$

The same helicity dependent splitting holds for the optical force, ${\overrightarrow{F}}_{rad}^{\mathrm{1,2}}={\overrightarrow{F}}_{rad}^{0}\pm {\overrightarrow{F}}_{rad}^{h}$ and for the optical torque, ${\overrightarrow{{\rm{\Gamma }}}}_{rad}^{\mathrm{1,2}}={\overrightarrow{{\rm{\Gamma }}}}_{rad}^{0}\pm {\overrightarrow{{\rm{\Gamma }}}}_{rad}^{h}$ (see Supplementary Information). In fact, we can generalize the Marston and Crichton result^46^ for a chiral sphere by considering the torque, ${\overrightarrow{{\rm{\Gamma }}}}_{rad}^{\mathrm{1,2}}=\pm \,{\tilde{\sigma }}_{abs}^{\mathrm{1,2}}{I}_{0}/\omega$, in terms of the chiral absorbtion cross section, ${\tilde{\sigma }}_{abs}^{\mathrm{1,2}}$, light intensity, *I*_0_, and frequency, *ω*. Thus, while for a non-chiral spherical particle the optical torque by a linearly polarized plane wave is zero, for a chiral sphere it is non-zero and it is related to the different absorption cross sections for LCP and RCP light.

**Figure 1:**
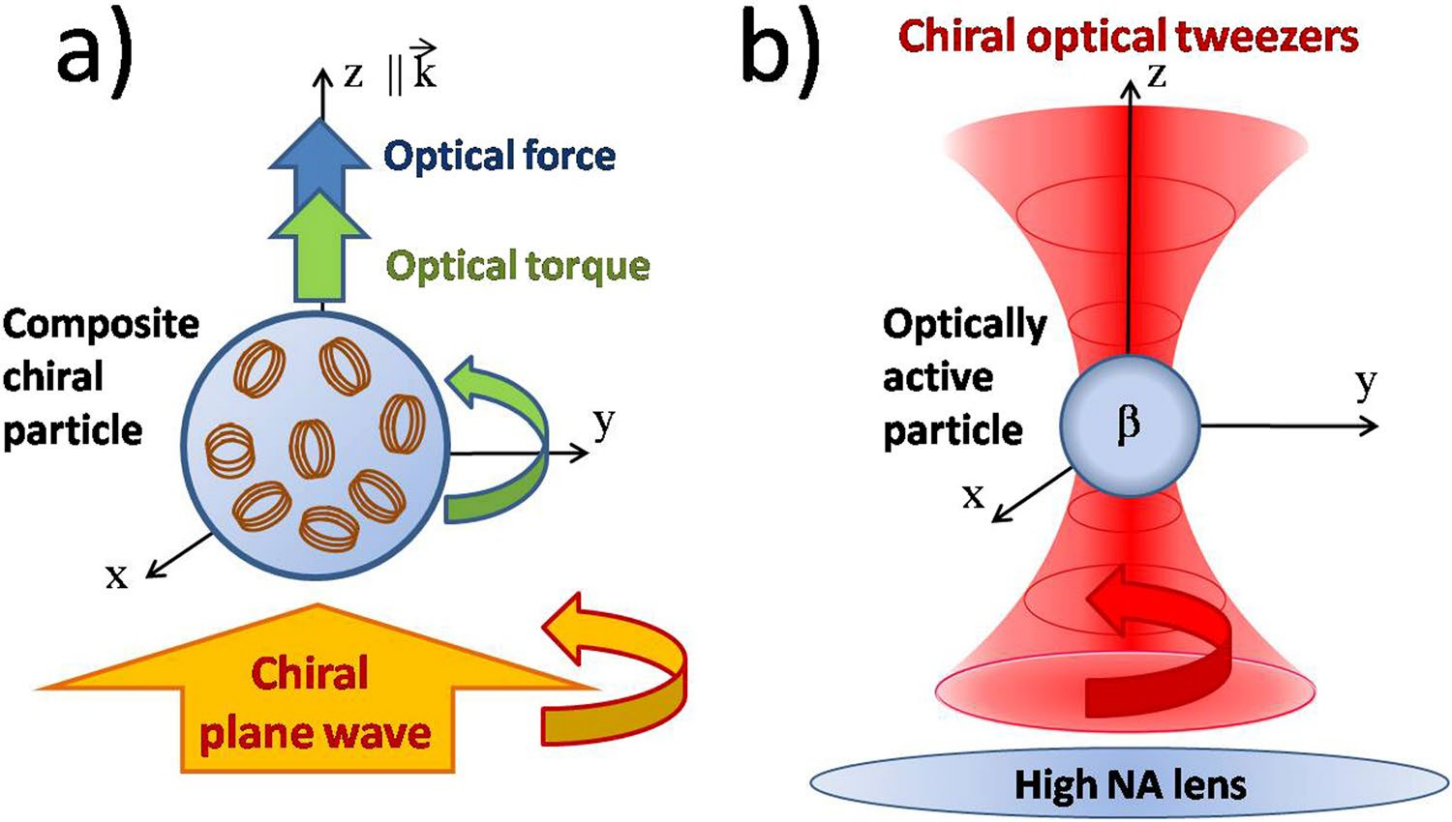
We investigate chiral optical forces in two different configurations. In (**a**) we investigate the optical force of a chiral plane wave incident on a composite chiral sphere made of an epoxy resin with embedded copper-coated stainless steel helices^47–49^. In (**b**) a chiral Gaussian beam is tightly focused through a high numerical aperture lens (chiral optical tweezers) and the optical trapping behaviour of an optically active particle is investigated.

**Figure 2:**
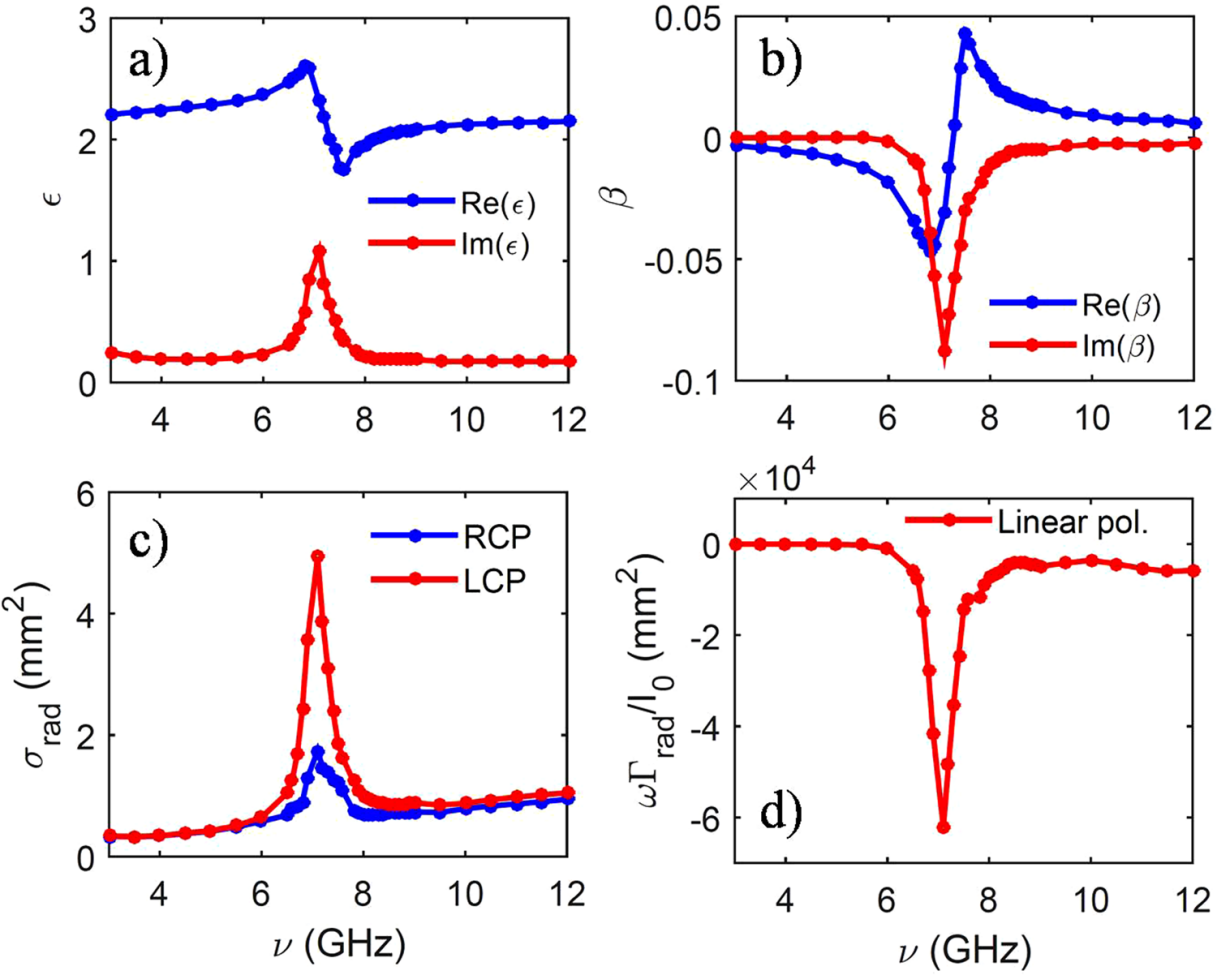
Optical properties and mechanical effects of light for chiral composites made of copper-coated stainless steel helices embedded in an epoxy resin^47^. (**a**) Real (blue) and imaginary (red) part of the electric permittivity. (**b**) Real (blue) and imaginary (red) part of the associated chirality parameter. (**c**) Radiation pressure cross section for a spherical particle with a radius of 2 mm and for different incident polarization, RCP (blue lines) and LCP (red lines). Note how a chiral gap opens in the radiation force at about 6 GHz related to the resonance of the chiral helices. (**d**) Frequency dependence of the optical torque, *ω*Γ_*rad*_/*I*_0_, for linear incident polarization related to the different absorption of LCP and RCP light by the chiral spherical particle.

We apply this theoretical approach to the case of a plane wave incident on a chiral spherical particle with a radius of 2 mm consisting of randomly oriented copper-coated stainless steel helices embedded in an epoxy resin^47,48^ with a volume fraction of 0.3% (see the sketch in Fig. 1a). This is a composite material that exhibits a frequency dependent chiral behaviour^47,49^. In Fig. 2a we show the effective dielectric constant of the composite material as obtained from combining the measured dielectric constant of the epoxy resin and the calculated dielectric constant of the chiral inclusions obtained from the scattering amplitude^47^ (see Supplementary Information for details). Instead in Fig. 2b we show the chirality parameter *β* in the microwave range as obtained from the work by Luebbers *et al*.^47^. Starting from these parameters, we show in Fig. 2c the calculated ${\tilde{\sigma }}_{rad}$ in a spectral range around 6 GHz where the imaginary part of *β* has a resonance. Due to the spherical symmetry of the particle, the only component of ${\overrightarrow{F}}_{rad}$ different from zero is in the direction of the incident field, *i.e*, a longitudinal radiation pressure. A chiral gap opens in the radiation cross section, and hence in the optical force, around the resonance at 6 GHz. Thus, we can directly observe that in a chiral material the amplitude of the optical forces is directly linked to its dichroism.

In Fig. 2d, we also show results related to the optical torque, Γ_*rad*_*ω*/*I*_0_, along the propagation, *z*, axis and for an incident linearly polarized light, that is a superposition of LCP and RCP light. Since LCP and RCP have different absorption and the optical torque is a consequence of the absorption of circularly polarized photons, we observe that a dichroic absorption spectrum directly reflects the performance of the spectrum of optical forces and torques in that range of frequencies.

### Chiral optical tweezers

We now consider the case of chiral optical tweezers, *i.e*., the optical trapping behaviour of an optically active particle in a tightly focused laser beam (see sketch in Fig. 1b). The starting point of our calculations is the angular spectrum representation^50^ of the focal fields generalized for a chiral Gaussian beam incident on a high numerical aperture objective lens^9,33,51^. In particular, we consider an incident laser beam propagating along the *z*-axis (see Fig. 1b), with 10 mW of power at *λ* = 632 nm and a Gaussian profile. The beam is focused through an aplanatic lens, with numerical aperture NA = 1.2 and filling factor of 2, that is the waist, *w*_0_, of the Gaussian beam is two times larger than the size of the back aperture of the (realistic) aplanatic lens^33^ (see section S3 of the Supplementary Information). The radiation force and torque acting on the particle are obtained by integrating the Maxwell stress tensor within the T-matrix formalism^33,52–54^:

18 $${\overrightarrow{F}}_{rad}={\oint }_{S}\langle {{\rm{T}}}_{{\rm{M}}}\rangle \cdot \hat{n}\,{\rm{d}}S,\,{\overrightarrow{{\rm{\Gamma }}}}_{rad}=-\,{\oint }_{S}(\langle {{\rm{T}}}_{{\rm{M}}}\rangle \times \overrightarrow{r})\cdot \hat{n}\,{\rm{d}}S,$$

where $\overrightarrow{r}$ is the vector position, $\hat{n}$ is the outward normal unit vector, 〈T_M_〉 is the averaged Maxwell stress tensor in the Minkowski form^55^ that describes the mechanical interaction of light and matter^9^, and the integration is carried out over a closed surface *S* surrounding the scattering particle. The Minkowski form of the Maxwell stress tensor is consistent with a choice of the Minkowski-type definition of light momenta in a medium as opposite to the Abraham-type one. While the Abraham-Minkowski dilemma has been going on for quite some time^55^, a possible solution has been considered^56^ where the Abraham and Minkowski momenta are two different ones, namely the kinetic momentum, where Abraham’s theory is concerned, and the canonical momentum, where Minkowski’s theory is concerned. Recently, the dilemma has been also discussed in the context of complex media^57,58^, where it was shown that while the Abraham-type quantities describe the energy flux and the group velocity of the wave, the Minkowski-type quantities describe the actual momentum and angular momentum carried by the wave^57,58^. However, no experiment has given final evidence in favour of one form or the other. Since most results in optical trapping and manipulation do not depend qualitatively on the momenta definition, we consistently employ the Minkowski momenta definition, which in fact are the ones most often employed for optical tweezers^9^.

Since we are interested in studying the chirality-dependent dynamics in the trap, we consider the typical case of a particle made with a material that exhibits optical activity in the visible, for this reason we chose a nanosphere with radius *a* = 200 nm immersed in water (*n*_m_ = 1.33) with an average refractive index *n*_p_ = 1.5 and the chiral parameter, *β* = 0.05. These values are operational values that are not strictly connected to real experimental parameters, but they are consistent with values for composite particles similar to the ones studied in Fig. 2. The dynamic quantities, ${\overrightarrow{F}}_{rad}$ and ${\overrightarrow{{\rm{\Gamma }}}}_{rad}$, are calculated starting from their definitions based on the Maxwell stress tensor^33,59^. In particular, we need to consider the multipole amplitudes ${\tilde{W}}_{lm}^{(p)}$ of a tightly focused beam. The expansion of a focused beam around the focal point is obtained by exploiting the angular spectrum representation^9,33,50,51^ (see section S3 in the Supplementary Information).

The expression for the radiation force along the direction of a unit vector $\hat{u}$, *i*.*e*., ${F}_{rad,\hat{u}}={\overrightarrow{F}}_{rad}\cdot \hat{u}$ can be obtained through the knowledge of the scattered amplitudes ${\tilde{A}}_{lm}^{(p)}$ related to the incident focal fields through the particle T-matrix. We can then plot the radiation force as a function of particle displacement in each spatial direction, *x*, *y*, and *z*. Since the trapping position of the particle in the axial (*z*) direction is typically offset from the centre of the coordinate system (taken at the nominal focus of the lens) due to the offset of the scattering force, we calculate the transverse forces at the equilibrium position, that is the *z* coordinate at which the axial force vanishes. To better compare optical forces on different systems, it is generally convenient to plot the dimensionless force efficiencies along the three cartesian directions, *Q*_*i*_ = *cF*_*i*_/*n*_m_*P* with *i* = *x*, *y*, *z*, *c* the light velocity in vacuum, and *P* the incident laser power.

**Figure 3:**
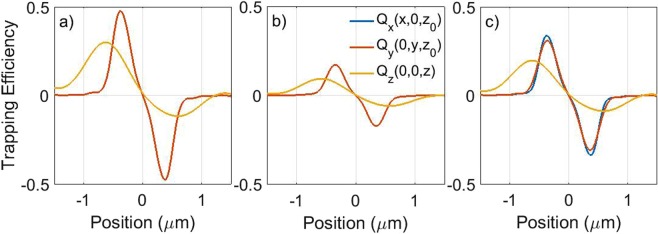
Optical trapping efficiencies for a chiral nanoparticle (*a* = 200 nm) along the focal region axes for LCP (**a**), RCP(**b**) and linear polarization (**c**). In the case of chiral field we have that *Q*_*x*_ = *Q*_*y*_, while for a linearly polarized incident beam a polarization anisotropy is evident that is related to the diffraction limited focusing^33^. Optical trapping appears to be much stronger for the LCP beam, as we expect for a left-chiral (positive *β*) nanoparticle.

In Fig. 3, we report the computed components of the optical trapping efficiencies, *Q*_*i*_, around the focal region, for different state of polarization of the incident field. As expected, the plots have a relative maximum and minimum approximately at the particle radius. In fact, at these points the greatest optical force is exerted because the sphere is located in the maximum gradient of intensity. This is consistent with the picture of the *optical gradient force* in approximated optical trapping regimes for particles much smaller or much larger than the light wavelength^9,60^. In proximity of the trapping point the radiation force can be linearized as a harmonic restoring force:

19 $$(\begin{array}{lll}{F}_{rad,x}(x,\,0,\,{z}_{0}) & = & -{\kappa }_{x}x\\ {F}_{rad,y}(0,\,y,\,{z}_{0}) & = & -{\kappa }_{y}y\\ {F}_{rad,z}(0,\,0,\,z) & = & -{\kappa }_{z}(z-{z}_{0})\end{array}$$

Thus, optical tweezers are approximated with an effective harmonic potential with spring constants or trap stiffnesses *κ*_*x*_, *κ*_*y*_*κ*_*z*_. These quantities are important because are the measured quantities when performing optical tweezers calibration^61^. To calculate the optical trap stiffnesses, we calculate the slope of the force-position plots at the equilibrium position, where the force vanishes.

**Figure 4:**
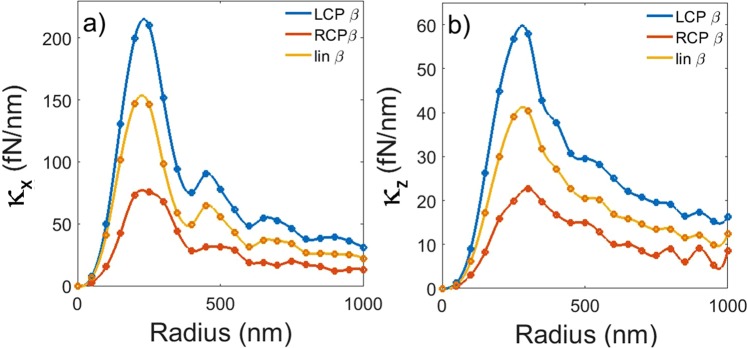
Transverse (**a**) and axial (**b**) optical trapping stiffnesses as a function of the particle radius for 10 mW incident power. Different polarization states result in different optical trapping forces. The siffnesses for LCP light (blue) are about three times larger than the ones RCP illumination (red). For linear polarization (yellow) optical trapping stiffnesses lie in between the circular polarization results.

At this stage, it is very interesting to investigate the dependence of the trap stiffnesses on the dimension of the chiral particle, *i.e*., the size-scaling of chiral optical trapping. The results of this study are reported in Fig. 4, where it is possible to compare the trap stiffnesses for different polarization states of the incident field. We note that in non-chiral (*β* = 0) cases the optical forces, and hence the trap stiffnesses, are the same for LCP and RCP radiation (see Fig. S2 and Sect. S2 of the Supplementary Information) and all equations then reduce to the standard Mie scattering. In contrast, for right-chiral particles with a positive chiral parameter (*β* = 0.05), the stiffness values are systematically higher for the LCP polarization. Thus, this indicates that the optical tweezers are an excellent experimental tool to discriminate the chiral nature of a particle of any size. Moreover, at a fixed wavelength, there is the possibility to optimize the trapping efficiency choosing the appropriate dimension for the investigated particle. For the case under study, we found the *κ*-values at about *a* ≈ 300 nm are up to four times higher than those obtained for *a* > 500 nm. This optimum value is related to the tight focusing used in optical tweezers that maximizes the optical force density when the particle size is of the order of the diffraction limited laser spot. We finally note that the modulation observed in the stiffnesses for larger particle size is explained in terms of interference between the different multipoles occurring in the scattering process^62,63^.

**Figure 5:**
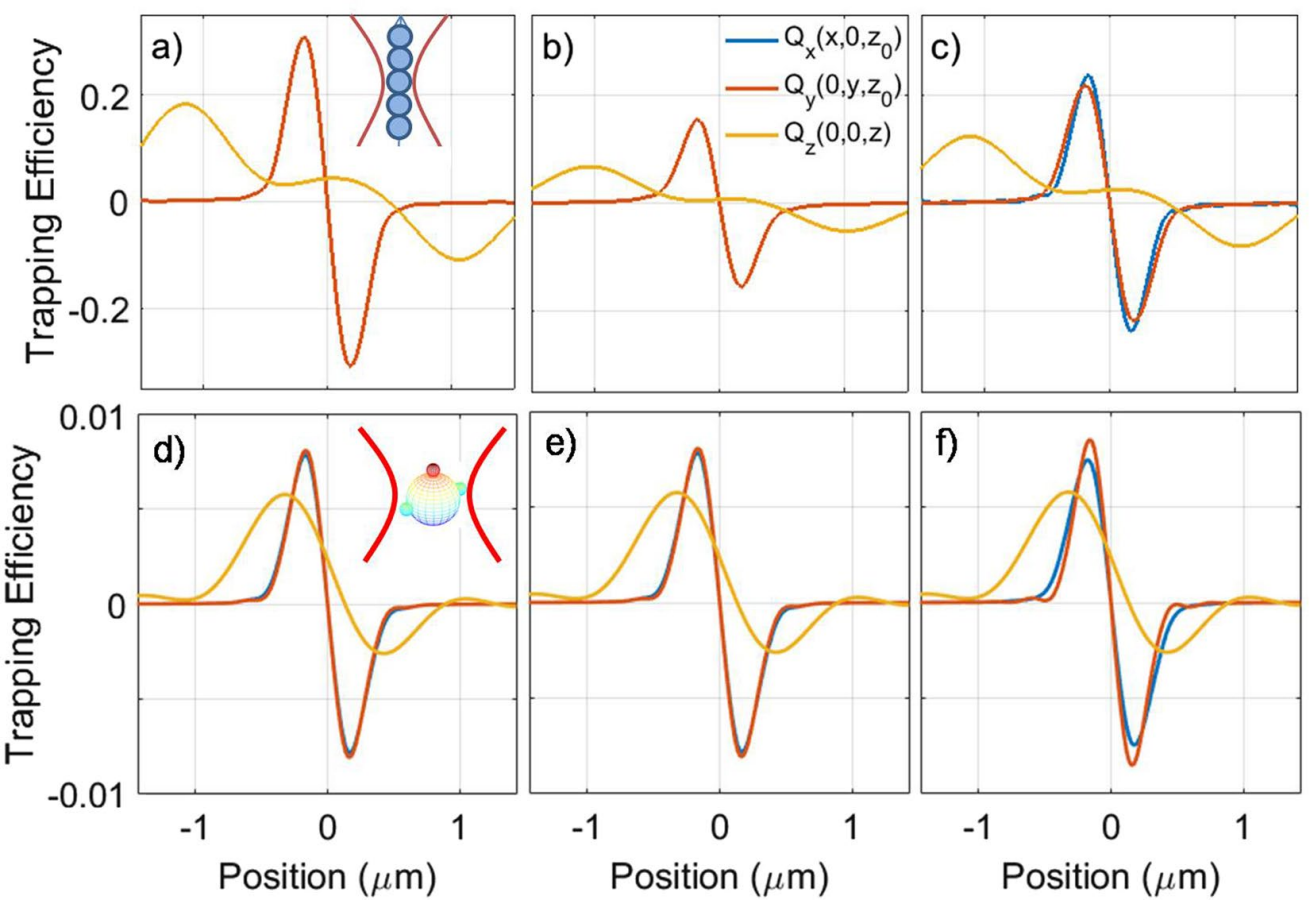
(**a**–**c**) Transverse (red, blue lines) and axial (yellow) optical trapping efficiencies for a model chiral nanowire for LCP (**a**), RCP (**b**), and linear (**c**) polarization. The optical activity of the nanowire yields the different optical forces for the different polarization states. For circularly polarized light the components in *x*,*y* are superposed because of symmetry. Instead for linear polarization the polarization splitting is evident due to the diffraction limited focusing^33,52,68^. (**d**–**f**) Transverse (red, blue lines) and axial (yellow) optical trapping efficiencies for a cluster with optical properties corresponding to the amino acids in the Murchison meteorite^35,37^ and a geometry sketched in the inset of (**d**). Results are shown for LCP (**d**), RCP (**e**), and linear (**f**) polarization. Note how the axial equilibrium point (when the force is zero), *z*_0_, is shifted from the nominal focus of the objective lens (origin of the *z* axis) because of the increased scattering force related to photon absorption.

In order to show the flexibility of the approach, we study two examples of chiral optical tweezers applied to anisotropic particles: a chiral nanowire and a particle cluster with properties that correspond to the amino acids discovered in the Murchison meteorite^35,37^. We extend the theory of chiral optical forces to non-spherical particles exploiting the cluster model as developed in the T-matrix approach^64–67^. We first use the same material properties as the spherical case and we consider a nanowire with a length of 2 *μ*m and a transverse size of 400 nm, modeled as a chain of 5 spheres with *a* = 200 nm radius (see the sketch in the inset of Fig. 5a). Figure 5a–c show the calculated trapping efficiencies for LCP (a), RCP (b), and linear (c) polarization. The decrease in the slope, *i.e*. the trap stiffness, of the nanowire axial trapping efficiency is apparent and it is related to the extended shape of the particle. This is expected as it has been observed^68,69^ and modeled in non-chiral nanowires^52,54^. The chiral nature of the nanowire yields a very different optical trapping force for the different polarization of the incident light in both the transverse and the axial directions.

Finally, we consider a model cluster with optical properties corresponding to the amino acids discovered in the Murchison meteorite^35,70,71^. These carbonaceous chondrites contain organic matter and have a huge significance in connection with the hypotheses of the extraterrestrial formation of prebiotic molecules and the origin of homochirality in organic matter^72–75^. In an astrophysical context it has also been pointed out that the near-field generated by a solar (blackbody) spectrum around interplanetary dust might generate optical trapping forces for atoms or small particles^76^. Thus, local near-field depolarization^73–75^ might produce chiral optical forces that could influence the sticking of adatoms or small particles to yield chiral molecules or structures. Furthermore, optical tweezers might have interesting applications as non-destructive contactless tools for the capture of interplanetary, cometary, or atmospheric dust *in situ*^77^. Indeed, sophisticated versions of optical tweezers (that can be modeled with the T-matrix approach) based on complex beams^78,79^ could be employed in the near future for interstellar dust or planetary rover missions. Thus, having a better knowledge on the optomechanical properties of exemplar dust particles might give a more precise idea on what to expect in those contexts and help engineering the optical fields required to distinguish optically active particles through their optomechanical interactions.

Here, we consider a simple model cluster made of a central sphere with a radius of 200 nm, surrounded by three smaller spheres with a radius of 50 nm. The cluster geometry is sketched in the inset Fig. 5d. All spheres composing the cluster have complex refractive indices, *n*_*R*_ = 1.5500000 ± 0.0006004*i* and *n*_*L*_ = 1.5500338 ± 0.0006000*i*, which are typical for the amino acids discovered in the Murchison meteorite^35,37^. These refractive indices correspond to a complex chiral parameter whose real and imaginary part are R*e*{*β*} ≈ 7 × 10^−6^ and I*m*{*β*} ≈ −3.2 × 10^−4^. Figure 5d–f show the calculated trapping efficiencies for LCP (d), RCP (e), and linear (f) polarization. We observe that, although the cluster is stably trapped, the equilibrium position for which the net force is zero along the beam propagation direction (*z*) is shifted with respect to the nominal focal point at *z*_0_ = 142 nm. This is the consequence of an increased radiation pressure due to light absorption and related to the occurrence of an imaginary part in the refractive indices. The cluster geometry also yields a small anisotropy in the optical force in the transverse (*x* − *y*) plane. However, the chiral behaviour is not very prominent since the real part of the chiral parameter is quite small. On the other hand, the presence of an imaginary part of the refractive index is also responsible for the transfer of angular momentum to the cluster. When trapped at its equilibrium position, *z*_0_, for LCP (*η* = 1) and RCP (*η* = 2) light the optical torque on the cluster is ${{\rm{\Gamma }}}_{rad}^{\mathrm{1,2}}\approx \pm 3.8\times {10}^{-2}$ pN ⋅ *μ*m. Because of the chiral nature of the cluster, for linearly polarized light we also obtain a small optical torque of about −1.3 × 10^−6^ pN ⋅ *μ*m (as for the case of Fig. 2d).

## Discussion

In conclusion, we explored the connection between optical activity and optical forces in a general light scattering framework. We used the T-matrix formalism to enlighten the relation between chiral fields and observable cross sections. Thus, we applied this general formalism to study optical forces on an exemplar complex spherical resin particle with inner copper-coated stainless steel helices, and to the important case of optical tweezers on chiral particles of different shape and composition. In all cases, a chiral gap opens in the optical forces giving clear evidence of the connection between chiral optomechanics and optical activity. We note that modeling more complex supramolecular chiral structures requires additional complexity because they cannot simply be considered in terms of an isotropic effective chiral permittivity or chiral parameter as that would not account for the Bragg reflection and chiral rotation^30,32^. While a simple core-shell model with a fictitious isotropic complex permittivity can account for some optomechanical properties, this involves an imaginary part, related to absorption, which is not realistic for these non-absorbing materials. A more realistic model should take the anisotropic nature of the material into account, for example by considering a multishell particle with alternating layers with different permittivities and chiral parameter or by coarse-graining the spherical particle into a globular cluster of birefringent particles whose optical axis rotates radially mimicking the orientation of the cholesteric liquid crystal molecules in the supramolecular structure. The examples discussed here represent a starting point for more complex calculations on non-spherical, complex chiral particles in chiral and super-chiral optical fields^24,80^ that can be tightly confined as in optical tweezers or extended as the fields used in speckle optical tweezers^61,81^. Mechanical effects of light are a tool to study chirality that can complement gyrotropic investigations or circular dichroism spectroscopy. In particular, optical tweezers offer the opportunity to investigate how chirality influences optomechanical phenomena such as optical forces, optical sorting, and light-driven rotations. The important goal of enantiomer separation by optical forces holds key applications in chemistry and pharmaceutics.

## Methods

The general light scattering theory in the T-matrix framework is based on the multipole expansion of incident, internal, and scattered fields and the application of the customary boundary conditions^8^ (See also Supplementary Information). Within this framework, cross sections and anisotropy are calculated, while optical forces^33^ and torques^59^ are obtained by applying conservation laws and the consequent integration of the Maxwell stress tensor on a surface surrounding the scattering particle^9^. For the case of optical tweezers, we generalized the angular spectrum representation of Richards and Wolf ^50,51^ to chiral fields and then calculated the optical tweezers stiffness for each particle or cluster^9,33^.

As it is typical for the solution of the light scattering problem for a sphere or a cluster and the evaluation of its T-matrix and optical forces, we calculate the relevant quantities through the inversion of a complex matrix that has infinite elements. Thus, a truncation of the multipolar expansion to some finite multipole order *l*_T_ has to be operated^8^. This is chosen to ensure the numerical stability of the calculated observables (e.g., cross-sections, optical forces, and optical torques). In practice, we check computationally for the existence of a minimum *l*-value, *l*_M_, such that when *l*_T_ > *l*_M_ the calculated quantities do not change within the numerical accuracy. The morphology of the modeled particle has crucial implications. In fact, for a cluster of *N* spheres, a matrix of order *dim* = 2*Nl*_T_(*l*_T_ + 2) needs to be inverted and the truncation order to be considered depends crucially on the geometrical packing in the aggregate^8^. Practically, we consider the smallest sphere of radius *R*_*cluster*_ that includes the whole aggregate with a corresponding size parameter *x*_*cluster*_ = *k*_m_*R*_*cluster*_ and truncate the expansion at *l*_T_ > *x*_*cluster*_. In our calculations we always verify that all our results are convergent within 0.1%. The force calculations can be generally performed on a standard PC. The calculations of the focal fields may require additional RAM to deal with large values of *l* that might be required for large spheres or clusters.

## Electronic supplementary material

Supplementary Information
