# Supplementary material for: Chiral optical tweezers for optically active particles in the T-matrix formalism
Source: Sci Rep. 2019 Jan 10;9:29. doi: 10.1038/s41598-018-36434-9 (PMC6328542; doi:10.1038/s41598-018-36434-9)
Supplement: Supplementary file 1 — Supplementary Information [file 41598_2018_36434_MOESM1_ESM.pdf]

Chiral optical tweezers for optically active particles in the T-matrix formalism

F. Patti[1,2], R. Saija[1,2], P. Denti[2], G. Pellegrini[3], P. Biagioni[3], M. A. Iatì[1,*], O. M. Maragò[1,*]

[1]CNR-IPCF, Istituto per i Processi Chimico-Fisici, I-98158 Messina, Italy.
[2]Dipartimento di Scienze Matematiche e Informatiche, Scienze Fisiche e Scienze della Terra, Università di Messina, I-98166 Messina, Italy.
[3]Dipartimento di Fisica, Politecnico di Milano, I-20133 Milano, Italy.

S1. Detailed analytical theory

We give here the detailed analytical theory. As explained in the main text, we consider an optically active particle with an average refractive index $n_{\mathrm{p}} = \sqrt{\epsilon/\mu}$ immersed in a non-dispersive, homogeneous medium of refractive index n_{m}. We use the Drude-Born-Fedorov (DBF) constitutive relations:[1,2]

$$\vec{D} = \epsilon\vec{E} + \frac{\alpha\epsilon}{k}\vec{\nabla}\times\vec{E}$$

$$\vec{B} = \mu\vec{H} + \frac{\beta\mu}{k}\vec{\nabla}\times\vec{H}$$

with the Maxwell's equations. If time symmetry is imposed: $\alpha = \beta$. The chirality parameter β is adimensional and it is related to the chiral refractive indices, $n_L = \bar{n}/(1-\beta\bar{n})$ and $n_R = \bar{n}/(1+\beta\bar{n})$, for left and right circularly polarized waves, respectively, where $\bar{n} = n_{\mathrm{p}}/n_{\mathrm{m}}$ is the average refractive index relative to the medium:

$$\beta = \frac{1}{2}\left(\frac{1}{n_R} - \frac{1}{n_L}\right).$$

*Email: mariaantonia.iati@cnr.it, onofrio.marago@cnr.it

In absence of charges and currents, we want to obtain equations similar to those of Helmholtz for a homogeneous and isotropic medium. The possibility to obtain, also in the chiral case, this kind of equations is a matter of primary importance, since it allows us to solve the scattering problem within the T-matrix formalism.

Using the DBF constitutive equations, an Helmholtz-type relation is found:

$$\nabla^2 \begin{bmatrix} \vec{E} \\ \vec{H} \end{bmatrix} + \mathcal{K}^2 \begin{bmatrix} \vec{E} \\ \vec{H} \end{bmatrix} = 0,$$

where $\mathcal{K}$ is the non-diagonal matrix:

$$\mathcal{K} = \frac{k}{1 - \beta^2 \mu\epsilon} \begin{pmatrix} \beta\mu\epsilon & i\mu \\ -i\epsilon & \beta\mu\epsilon \end{pmatrix}.$$

In this kind of medium the fields are coupled with each other during propagation. Bohren has shown that a good way to decompose this field is to consider any fields in the chiral medium as an overlap of circularly polarized fields. Formally we consider the linear transformation:

$$\begin{bmatrix} \vec{E} \\ \vec{H} \end{bmatrix} = \mathcal{A} \begin{bmatrix} \vec{Q}_L \\ \vec{Q}_R \end{bmatrix},$$

$$\mathcal{A} = \begin{pmatrix} 1 & -i/n_{\mathrm{p}} \\ -in_{\mathrm{p}} & 1 \end{pmatrix},$$

where:

$$n_{\mathrm{p}} = \sqrt{\frac{\epsilon}{\mu}}.$$

Thus, inside a chiral particle, the internal fields are expressed in terms of the chiral fields:

$$\begin{cases} \vec{E}_P = \vec{Q}_L - \frac{i}{n_{\mathrm{p}}} \vec{Q}_R \\ \vec{H}_P = -in_{\mathrm{p}} \vec{Q}_L + \vec{Q}_R. \end{cases}$$

Expanding in multipole fields we have that:

$$\begin{cases} \vec{Q}_L = \sum_{lm} C_{Llm} [\vec{J}^{(1)}_{Llm} + \vec{J}^{(2)}_{Llm}] \\ \vec{Q}_R = \sum_{lm} C_{Rlm} [-\vec{J}^{(1)}_{Rlm} + \vec{J}^{(2)}_{Rlm}], \end{cases}$$

with the left-handed multipoles defined as:

$$\vec{J}^{(1)}_{Llm} = j_l(k_L r) \vec{X}^{(1)}_{lm}(r),$$

$$\vec{J}^{(2)}_{Llm} = \frac{1}{k_L}\vec{\nabla} \times \vec{J}^{(1)}_{Llm},$$

where $k_L = n_L k$, and with a similar definition for the right-handed multipoles, $\vec{J}^{(1)}_{Rlm}$ and $\vec{J}^{(2)}_{Rlm}$. We used J-multipole fields to ensure the regularity of the fields everywhere within the sphere. The coefficients C_{Llm} and C_{Rlm} are determined by imposing the boundary conditions across the surface of the sphere. We assume that the surrounding medium is homogeneous, isotropic, and nonmagnetic, and that the incident field and the scattered field can be written as:

$$\vec{E}_i = E_0 \sum_{plm} \vec{J}^{(p)}_{lm}(\vec{r}, k) W^{(p)}_{lm},$$

$$\vec{E}_s = E_0 \sum_{plm} \vec{H}^{(p)}_{lm}(\vec{r}, k) A^{(p)}_{lm},$$

while the magnetic field in the external region is given by

$$\vec{H} = -\frac{i}{k}\nabla \times \vec{E}.$$

Applying the boundary conditions for each l and m, we obtain:

$$h_l(x)A^{(1)}_{lm} + j_l(x)W^{(1)}_{lm} = j_l(x_L)C_{Llm} + \frac{i}{n_{\mathrm{p}}} j_l(x_R)C_{Rlm},$$

$$-\frac{1}{x}w'_l(x)A^{(2)}_{lm} - \frac{1}{x}u'_l(x)W^{(2)}_{lm} = -\frac{1}{x_L}u'_l(x_L)C_{Llm} + \frac{1}{x_R n_{\mathrm{p}}}u'_l(x_R)C_{Rlm},$$

$$\frac{in_{\mathrm{m}}}{x}w'_l(x)A^{(1)}_{lm} + \frac{in_{\mathrm{m}}}{x}u'_l(x)W^{(1)}_{i,lm} = \frac{in_{\mathrm{p}}}{x_L}u'_l(x_L)C_{Llm} - \frac{1}{x_R}u'_l(x_R)C_{Rlm},$$

$$-in_{\mathrm{m}}h_l(x)A^{(2)}_{lm} - in_{\mathrm{m}}j_l(x)W^{(2)}_{i,lm} = -in_{\mathrm{p}}j_l(x_L)C_{Llm} - j_l(x_R)C_{Rlm}.$$

In these formulas $j_l(x)$ and $h_l(x)$ are spherical Bessel and spherical Hankel functions, while $u_l(x) = xj_l(x)$ and $w_l(x) = xh_l(x)$ are Riccati-Bessel and Riccati-Hankel functions, the prime indicates differentiation, the variables $x = k_{\mathrm{m}}a$, $x_L = k_L a$, and $x_R = k_R a$ are size parameters with a being the radius of the particle, and $\bar{\mathrm{n}} = n_{\mathrm{p}}/n_{\mathrm{m}}$ is the relative average refractive index of the particle with respect to the refractive index of the medium, n_{m}. So we obtain the T-matrix as:

$$A^{(p)}_{lm} = -\sum_{p'} R^{(pp')}_l W^{(p')}_{lm}$$

$$T^{(pp')}_{lml'm'} = -R^{(pp')}_l \delta_{ll'}\delta_{mm'}$$

We can now write explicitly the elements of the T-matrix for the chiral sphere as:

$$R^{(11)}_l = \frac{1}{D_l}\{[n_\mathrm{m} u'_l(x_L)u(x) - u_l(x_L)u'(x)][u'_l(x_R)w_l(x) - n_\mathrm{m} u_l(x_R)w'_l(x)]$$

$$+[u'_l(x_L)w_l(x) - n_\mathrm{m} u_l(x_L)w'_l(x)][n_\mathrm{m} u'_l(x_R)u_l(x) - u_l(x_R)u'_l(x)]\},$$

$$R^{(12)}_l = R^{(21)}_l =$$

$$\frac{1}{D_l}\{[u'_l(x_L)u(x) - n_\mathrm{m} u_l(x_L)u'(x)][u'_l(x_R)w_l(x) - n_\mathrm{m} u_l(x_R)w'_l(x)]$$

$$-[u'_l(x_L)w_l(x) - n_\mathrm{m} u_l(x_L)w'_l(x)][u'_l(x_R)u_l(x) - n_\mathrm{m} u_l(x_R)u'_l(x)]\},$$

$$R^{(22)}_l = \frac{1}{D_l}\{[n_\mathrm{m} u'_l(x_L)w(x) - u_l(x_L)w'(x)][u'_l(x_R)u_l(x) - n_\mathrm{m} u_l(x_R)u'_l(x)]$$

$$+[u'_l(x_L)u_l(x) - n_\mathrm{m} u_l(x_L)u'_l(x)][n_\mathrm{m} u'_l(x_R)w_l(x) - u_l(x_R)w'_l(x)]\},$$

with:

$$D_l = [n_\mathrm{m} u'_l(x_L)w_l(x) - u_l(x_L)w'_l(x)][u'_l(x_R)w_l(x) - n_\mathrm{m} u_l(x_R)w'_l(x)]$$

$$+[u'_l(x_L)u_l(x) - n_\mathrm{m} u_l(x_L)u'_l(x)][n_\mathrm{m} u'_l(x_R)w_l(x) - u_l(x_R)w'_l(x)].$$

In the case of an achiral sphere, $\beta = 0$, applying the boundary conditions yields the Mie solution that is diagonal in the parity index, p:

$$T^{(pp')}_{lml'm'} = -R^{(p)}_l \delta_{ll'}\delta_{mm'}\delta_{pp'}$$

and the corresponding cross sections are:

$$\sigma_{ext} = \frac{2\pi}{k^2_\mathrm{m}} \sum_{pl} (2l+1)\,\mathrm{Re}\{R^{(p)}_l\},$$

$$\sigma_{scat} = \frac{2\pi}{k^2_\mathrm{m}} \sum_{pl} (2l+1)|R^{(p)}_l|^2.$$

Instead, in the chiral case, $\beta \neq 0$, we have that the corresponding cross sections are:

$$\tilde{\sigma}^\eta_{ext} = \frac{2\pi}{k^2_\mathrm{m}} \sum_{lpp'} (2l+1) R^{(pp')}_l [\delta_{pp'} - (-1)^\eta (1-\delta_{pp'})], \qquad (1)$$

$$\tilde{\sigma}^{\eta}_{scat} = \frac{2\pi}{k_{\rm m}^2} \sum_{lp} \sum_{p'p''} (2l+1) R_l^{pp'*} R_l^{pp''} [\delta_{p'p''} - (-1)^{\eta}(1-\delta_{p'p''})], \tag{2}$$

where $\eta = 1$ corresponds to left-handed polarization, $+$, *i.e.*, to a polarization unit vector $\hat{c}_1 = (\hat{x} + i\hat{y})/\sqrt{2}$, while $\eta = 2$ corresponds to a right-handed polarization, $-$, *i.e.*, to a polarization unit vector $\hat{c}_2 = (\hat{x} - i\hat{y})/\sqrt{2}$. It should be noted here that the elements of the matrix $R_l^{(pp')}$ for a chiral particle cannot be described in terms of Mie coefficients (a_l, b_l). We can also consider the expansion for the chiral *asymmetry parameter*:

$$\begin{aligned} \tilde{\sigma}^{\eta}_{scat} \tilde{g}_i{}^{\eta} = \frac{4\pi}{k_{\rm m}^2} \sum_{lp} \mathrm{Re} \Bigg\{ \frac{l(l+2)}{l+1} \sum_{p'p''} R_l^{(pp')} R_{l+1}^{(pp'')*} [\delta_{p'p''} - (-1)^{\eta}(1-\delta_{p'p''})] \\ + \frac{2l+1}{l(l+1)} \sum_{p''p'''} R_l^{(pp''')} R_{l+1}^{(p'p''')*} [1-\delta_{pp'}][\delta_{p''p'''} - (-1)^{\eta}(1-\delta_{p'p''})] \Bigg\}. \end{aligned} \tag{3}$$

We calculate explicitly the various cross sections. From Eq.(1) we have the extinction cross sections:

$$\tilde{\sigma}^{1}_{ext} = \frac{2\pi}{k_{\rm m}^2} \sum_{l} (2l+1)\, \mathrm{Re}\{R_l^{(11)} + R_l^{(22)} + 2R_l^{(12)}\},$$

$$\tilde{\sigma}^{2}_{ext} = \frac{2\pi}{k_{\rm m}^2} \sum_{l} (2l+1)\, \mathrm{Re}\{R_l^{(11)} + R_l^{(22)} - 2R_l^{(12)}\}.$$

So we can separate the extinction cross section into two parts: one that is helicity independent and one that is helicity dependent.

$$\tilde{\sigma}^{1,2}_{ext} = \tilde{\sigma}^{0}_{ext} \pm \tilde{\sigma}^{h}_{ext},$$

with:

$$\tilde{\sigma}^{0}_{ext} = \frac{2\pi}{k_{\rm m}^2} \sum_{l} (2l+1)\, \mathrm{Re}\{R_l^{(11)} + R_l^{(22)}\},$$

$$\tilde{\sigma}^{h}_{ext} = \frac{2\pi}{k_{\rm m}^2} \sum_{l} 2(2l+1)\, \mathrm{Re}\{R_l^{(22)}\}.$$

Instead from Eq. (2):

$$\begin{aligned} \tilde{\sigma}^{1}_{scat} = \frac{2\pi}{k_{\rm m}^2} \sum_{l} (2l+1) \Big\{ |R_l^{(11)}|^2 + |R_l^{(22)}|^2 + 2|R_l^{(12)}|^2 \\ + \Big[R_l^{(11)*} R_l^{(12)} + 2|R_l^{(12)}|^2 + R_l^{(22)*} R_l^{(12)} \Big] \Big\}, \end{aligned}$$

$$\tilde{\sigma}^2_{scat} = \frac{2\pi}{k_{\rm m}^2}\sum_l (2l+1)\left\{|R_l^{(11)}|^2 + |R_l^{(22)}|^2 + 2|R_l^{(12)}|^2 \right.$$
$$\left. - \left[R_l^{(11)*}R_l^{(12)} + 2|R_l^{(12)}|^2 + R_l^{(22)*}R_l^{(12)}\right]\right\}.$$

Also this relation can break into two parts:

$$\tilde{\sigma}^{1,2}_{scat} = \tilde{\sigma}^0_{scat} \pm \tilde{\sigma}^h_{scat},$$

where:

$$\tilde{\sigma}^0_{scat} = \frac{2\pi}{k_{\rm m}^2}\sum_l (2l+1)\left[|R_l^{(11)}|^2 + |R_l^{(22)}|^2 + 2|R_l^{(12)}|^2\right],$$

$$\tilde{\sigma}^h_{scat} = \frac{2\pi}{k_{\rm m}^2}\sum_l (2l+1)\left[R_l^{(11)*}R_l^{(12)} + 2|R_l^{(12)}|^2 + R_l^{(22)*}R_l^{(12)}\right].$$

It is then straightforward to obtain the absorption cross section since $\tilde{\sigma}^{1,2}_{abs} = \tilde{\sigma}^{1,2}_{ext} - \tilde{\sigma}^{1,2}_{scat} = \tilde{\sigma}^0_{abs} \pm \tilde{\sigma}^h_{scat}$, where:

$$\tilde{\sigma}^0_{abs} = \tilde{\sigma}^0_{ext} - \tilde{\sigma}^0_{scat},$$

$$\tilde{\sigma}^h_{abs} = \tilde{\sigma}^h_{ext} - \tilde{\sigma}^h_{scat}.$$

Finally, from Eq. (3) the asymmetry parameter term, that is important for the calculation of the optical force, can also be calculated:

$$\tilde{\sigma}^{1,2}_{scat}\tilde{g_i}^{1,2} = \gamma^0 \pm \gamma^h,$$

with:

$$\gamma^0 = \frac{4\pi}{k_{\rm m}^2}\sum_l \mathrm{Re}\left\{\frac{l(l+2)}{l+1}\left[R_l^{(11)}R_{l+1}^{(11)*} + R_l^{(22)}R_{l+1}^{(22)*} + 2R_l^{(12)}R_{l+1}^{(12)*}\right] + \right.$$
$$\left. \frac{2(2l+1)}{l(l+1)}\left[R_l^{(11)}R_l^{(12)*} + R_l^{(12)}R_l^{(22)*}\right]\right\},$$

and:

$$\gamma^h = \frac{4\pi}{k_{\rm m}^2}\sum_l \mathrm{Re}\left\{\frac{l(l+2)}{l+1}\left[R_l^{(11)}R_{l+1}^{(12)*} + R_l^{(12)}R_{l+1}^{(11)*} + R_l^{(22)}R_{l+1}^{(12)*} + R_l^{(12)}R_{l+1}^{(22)*}\right]\right.$$
$$\left. + \frac{2l+1}{l(l+1)}\left[R_l^{(11)}R_l^{(22)*} + |R_l^{(12)}|^2\right]\right\}.$$

At this point we can write the radiation pressure on the particle as:

$$\vec{F}_{rad} = \frac{n}{c} I_0 [\tilde{\sigma}_{ext} - \tilde{\sigma}_{scat} \tilde{g}_i] \hat{k}_0 = \frac{n}{c} I_0 \tilde{\sigma}_{rad} \hat{k}_0,$$

with $\tilde{\sigma}_{rad} = \tilde{\sigma}_{ext} - \tilde{\sigma}_{scat}\tilde{g}_i$. Therefore, we can separate the radiation pressure cross section, the optical force, and the optical torque as:

$$\tilde{\sigma}^{1,2}_{rad} = \tilde{\sigma}^{0}_{rad} \pm \tilde{\sigma}^{h}_{rad}, \quad \vec{F}^{1,2}_{rad} = \vec{F}^{0}_{rad} \pm \vec{F}^{h}_{rad}, \quad \vec{\Gamma}^{1,2}_{rad} = \vec{\Gamma}^{0}_{rad} \pm \vec{\Gamma}^{h}_{rad}.$$

S2. Radiation pressure cross-section for plane wave illumination

We consider some further implications for the specific case of a plane wave incident on a chiral sphere.

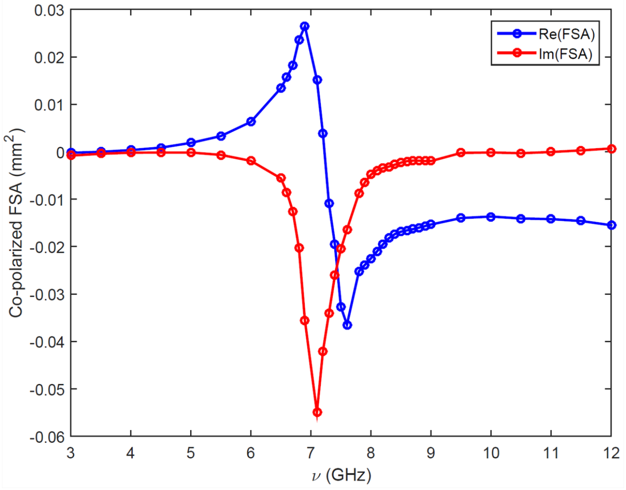

Figure S1: Co-polarized forward scattering amplitude (FSA) as obtained from Luebbers and co-workers[3] for a model chiral particle made of copper-coated stainless steel helices embedded in an epoxy resin.

As explained in the main text, the first sample we consider is the one realized by Luebbers and co-workers[3] that consists of copper-coated stainless steel right-handed helices embedded and randomly oriented in an EPOKWICK epoxy resin. The volume fraction of the inclusions is 0.3% and their number density is $\rho = 12.17 \times 10^6$ m^{-3}. Each coil consists of a copper-coated

stainless steel wire wound three times on itself. The density of the epoxy matrix is 1.15 g/cm^3. We assume a constant value for the complex permittivity of epoxy matrix as its frequency dependence in the $3-12$ GHz range is weak and can be neglected. Thus, we use the value of the permittivity measured[3,4] at 5 GHz, that is $\epsilon_{\rm epo} = 2.23 + i0.18$. The complex dielectric constant of the composite material, $\epsilon_{\rm p}$, is instead calculated by combining epoxy resin dielectric constant with the co-polarized forward scattering amplitude (FSA), $f_x = \vec{f} \cdot \hat{e}_x = f'_x + i f''_x$, obtained from Luebbers and co-workers[3] (see fig. S1) for an incident $x-$polarized plane wave through the expression:

$$\epsilon_{\rm p} = \epsilon_{\rm epo}\epsilon_{\rm hel} = \epsilon_{\rm epo}\left(1 + \frac{2\pi}{k^2}\rho f_x\right)^2 . \tag{4}$$

Where $\epsilon_{\rm h}$ is related to the helicoidal inclusions and its real and imaginary parts are related to the real and imaginary parts of the measured co-polarized forward scattering amplitude as:

$$\epsilon'_{\rm hel} = 1 + \frac{4\pi}{k^2}\rho f'_x + \frac{4\pi^2}{k^4}\rho^2 (f'^2_x - f''^2_x) \tag{5}$$

$$\epsilon''_{\rm hel} = \frac{4\pi}{k^2}\rho f''_x + \frac{8\pi^2}{k^4}\rho^2 f'_x f''_x . \tag{6}$$

Finally, the real, $\epsilon'_{\rm p}$, and imaginary, $\epsilon''_{\rm p}$, part of the effective dielectric constant of the composite material shown in fig. 2a and 2b are simply expressed as:

$$\begin{aligned} \epsilon'_{\rm p} &= \epsilon'_{\rm epo}\epsilon'_{\rm hel} - \epsilon''_{\rm epo}\epsilon''_{\rm hel} \\ \epsilon''_{\rm p} &= \epsilon'_{\rm epo}\epsilon''_{\rm hel} + \epsilon''_{\rm epo}\epsilon'_{\rm hel} . \end{aligned}$$

As an example, fig. S2a shows the volumetric dependence of the splitting between chiral optical forces with different polarization of the incident light at a fixed frequency of 6 GHz for spherical particles with optical response given by the composite material.

The second case we consider is a model chiral particle made of a material that exhibits optical activity in the visible, and more specifically we chose a nanosphere with radius a = 200 nm, refraction index 1.5, immersed in water, $n_{\rm m}$ = 1.33, and with a chiral parameter β = +0.05. Computing the radiation pressure cross sections, see fig. S2(b-c), as a function of the particle size (fig. S2b) and of the chirality parameter (fig. S2c) we can notice

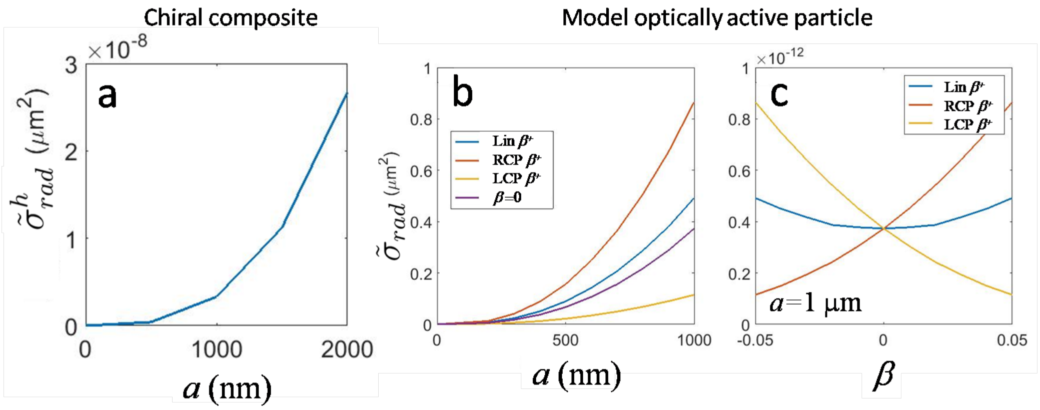

Figure S2: (a) Splitting of the radiation pressure cross section, for different helicity at 6 GHZ, as a function of particle radius, a. The model chiral particle is made of copper-coated stainless steel helices embedded in an epoxy resin. (b-c) Radiation pressure cross section for chiral (LCP: yellow, linear: red, RCP: light blue) and achiral (violet line) non-absorbing particles at $\lambda = 632$ nm as a function of (b) particle radius with fixed $\beta = +0.05$, and (c) chirality parameter with fixed radius $a = 1$ μm.

the difference between chiral and achiral interaction when illuminating with different polarization states. For the case of achiral particles ($\beta = 0$) the optical forces, and hence the trap stiffnesses, are the same for left and right circularly polarized radiation. All equations then reduce to the standard Mie scattering. In particular, figure S2b shows a comparison of the radiation pressure cross section as a function of the particle size for chiral ($\beta = +0.05$) and achiral ($\beta = 0$) particles and different polarization states. For the achiral case (violet line) the radiation pressure cross section is independent of the incident light polarization. In fact, in fig. S2c we show the radiation pressure cross section for a particle with fixed size as a function of the chiral parameter, β. Here the cross section curves for different polarization states collapse to the same value at $\beta = 0$, *i.e.*, for the achiral case.

S3. Radiation force and focused fields in the T-matrix formalism

A radiation field illuminating a particle yields a radiation force $\vec{F}_{rad}$ and a torque $\vec{\Gamma}_{rad}$, which contribute to its dynamics. As discussed in the main text, we can use conservation laws for linear and angular momentum in a

light scattering process to derive these optomechanical quantities. Thus, the time-averaged optical force and torque on a generic particle exerted by a monochromatic light are:[5–7]

$$\vec{F}_{rad} = \oint_S \langle \mathsf{T}_{\mathrm{M}} \rangle \cdot \hat{n} \; \mathrm{d}S \; , \quad \vec{\Gamma}_{rad} = -\oint_S (\langle \mathsf{T}_{\mathrm{M}} \rangle \times \vec{r}) \cdot \hat{n} \; \mathrm{d}S, \tag{7}$$

where the integration is carried out over a surface S surrounding the scattering particle, $\hat{n}$ is the outward normal unit vector, $\vec{r}$ is the vector position, and $\langle \mathsf{T}_{\mathrm{M}} \rangle$ is the averaged Maxwell stress tensor in the Minkowski form[8] describing the optomechanical interaction. Since we consider harmonic fields, at frequency ω in a homogeneous, linear, and non-dispersive medium, we can simplify[5] the general expression of $\langle \mathsf{T}_{\mathrm{M}} \rangle$ by considering the real physical fields, *e.g.*, $\vec{\mathcal{E}}(\vec{r}, t) = Re\left\{\vec{E}(\mathbf{r})e^{-i\omega t}\right\}$, in terms of the complex amplitudes, *e.g.* $\vec{E} = \vec{E}(\vec{r})$. So that:[5,6]

$$\langle \mathsf{T}_{\mathrm{M}} \rangle = \frac{\varepsilon_{\mathrm{m}}}{2} Re\left\{\vec{E} \otimes \vec{E}^* + \frac{c^2}{n_{\mathrm{m}}^2}\vec{B} \otimes \vec{B}^* - \frac{1}{2}\left(|\vec{E}|^2 + \frac{c^2}{n_{\mathrm{m}}^2}|\vec{B}|^2\right)\mathsf{I}\right\} \tag{8}$$

the fields, $\vec{E} = \vec{E}_{\mathrm{i}} + \vec{E}_{\mathrm{s}}$ and $\vec{B} = \vec{B}_{\mathrm{i}} + \vec{B}_{\mathrm{s}}$, are the total electric and magnetic fields, superposition of the incident $(\vec{E}_{\mathrm{i}}, \vec{B}_{\mathrm{i}})$ and scattered $(\vec{E}_{\mathrm{s}}, \vec{B}_{\mathrm{s}})$ fields, $\otimes$ is the dyadic product, and I is the dyadic unit.

The incident and scattered fields can be expanded in vector spherical harmonics, *e.g.*, as in Section S1. By substituting those expansions in terms of coefficients $W_{\mathrm{i},lm}^{(p)*}$ and $A_{\mathrm{s},lm}^{(p)*}$ and by taking the asymptotic limit $(r \to \infty)$, the general expression for the radiation force along the direction of a unit vector $\hat{u}$, *i.e.*, $F_{rad}(\hat{u}) = \vec{F}_{rad} \cdot \hat{u}$ is obtained as:[6]

$$F_{rad}(\hat{u}) = -\frac{\varepsilon_{\mathrm{m}} E_0^2}{2k_{\mathrm{m}}^2} Re\left\{\sum_{plm}\sum_{p'l'm'} i^{l-l'} I_{lml'm'}^{(pp')}(\hat{u}) \left[A_{\mathrm{s},lm}^{(p)*} A_{\mathrm{s},l'm'}^{(p')} + W_{\mathrm{i},lm}^{(p)*} A_{\mathrm{s},l'm'}^{(p')}\right]\right\} \; , \tag{9}$$

where the integrals $I_{lml'm'}^{(pp')}(\hat{u})$ are expressed in closed form in terms of spherical harmonics.[6]

This is the starting point to calculate the radiation force components in an optical tweezers. In this case we need first to calculate the multipole amplitudes $\tilde{W}_{\mathrm{i},lm}^{(p)}$ of a tightly focused beam. These are obtained by exploiting

the angular spectrum representation:[5,9,6]

$$\vec{E}_{\rm f}(x,y,z) = \frac{ik_{\rm t} f e^{-ik_{\rm t} f}}{2\pi} \int_0^{\theta_{\max}} \sin\theta \int_0^{2\pi} \vec{E}_{{\rm ff},t}(\theta,\varphi) e^{i[k_{{\rm t},x}x + k_{{\rm t},y}y]} e^{ik_{{\rm t},z}z} \, {\rm d}\varphi \, {\rm d}\theta \ , \quad (10)$$

where f is the focal length of the objective lens, $k_{\rm t}$ is the wavenumber transmitted through the lens, the polar angle θ is the deflection angle (related to the Abbe's sine condition[5]), and each transmitted plane wave, $\vec{E}_{{\rm ff},t}(\theta,\varphi)$, has an amplitude, $E_{{\rm ff},t}(\theta,\varphi)$, that can be calculated in terms of the incident field on the objective lens, $E_{\rm i}(\theta,\varphi)$, through an intensity law:[5]

$$E_{{\rm ff},t}(\theta,\varphi) = E_{\rm i}(\theta,\varphi)\sqrt{\frac{n_{\rm i}}{n_{\rm m}}}\sqrt{\cos\theta} f_w \quad (11)$$

where n_i is the refractive index before the lens principal plane, $n_{\rm m}$ is the one in the object space, and f_w is an apodization function[6] (or pupil function):

$$f_w = \exp\left(-\frac{\sin^2\theta}{f_0^2 \sin^2\theta_{\max}}\right) , \quad (12)$$

where the filling factor, $f_0 = w_0/(f\sin\theta_{\max})$, of a realistic objective lens[6] with numerical aperture NA$=n_{\rm i}\sin\theta_{\max}$, is simply the ration between the waist, w_0, of the incident Gaussian beam and the (effective) back aperture of the objective lens, $f\sin\theta_{\max}$. Note that when $w_0 \to \infty$, the apodization function $f_w = 1$ and we recover the original description of the transmitted fields by Richards and Wolf.[10]

The transmitted fields can be expanded into spherical Bessel multipoles around a point $\mathbf{P}$ displaced with respect to the nominal focal point $\mathbf{O}$, with expansion coefficients $\tilde{W}^{(p)}_{{\rm i},lm}(\mathbf{P})$. Therefore, the amplitudes of the focused field are:[5]

$$\tilde{W}^{(p)}_{{\rm i},lm}(\mathbf{P}) = \frac{ik_{\rm t} f e^{-ik_{\rm t} f}}{2\pi} \int_0^{\theta_{\max}} \sin\theta \int_0^{2\pi} E_{{\rm ff},t}(\theta,\varphi) \, W^{(p)}_{{\rm i},lm}(\hat{\mathbf{k}}_{\rm i},\hat{\mathbf{e}}_{\rm i}) \, e^{i\mathbf{k}_{\rm t}\cdot\mathbf{P}} \, {\rm d}\varphi \, {\rm d}\theta, \quad (13)$$

the amplitudes $\tilde{W}^{(p)}_{{\rm i},lm}(\mathbf{P})$, defining the focal fields, can be numerically calculated once the characteristics of the optical system are known. The expression for the radiation force along the direction of a unit vector $\hat{u}$, *i.e.*,

$F_{rad}(\hat{u}) = \vec{F}_{rad} \cdot \hat{u}$ can be obtained through the knowledge of the scattered amplitudes $\tilde{A}^{(p)}_{\mathrm{s},lm}$ related to the incident focal fields through the particle T-matrix. In practice, the expression of the optical force in the focal region is obtained from the correspondent one in Eq. (9) by changing $E_0 W^{(p)}_{\mathrm{i},lm} \to \tilde{W}^{(p)}_{\mathrm{i},lm}(\mathbf{P})$ and $E_0 A^{(p)}_{\mathrm{s},lm} \to \tilde{A}^{(p)}_{\mathrm{s},lm}$. Similar considerations apply for the calculation of the radiation torque.[5,6,11]

S4. Chiral optical tweezers for a chiral absorbing particle

Considering a laser with a power of 10 mW, a wavelength of 632 nm and a TEM_{00} Gaussian profile, focussed by a lens with a numeral aperture of 1.2, we considered the case of a particle with refractive index $n_{\mathrm{p}} = 1.5$, immersed in water, $n_{\mathrm{m}} = 1.33$, and with a complex chiral parameter, $\beta = 0.05 - 0.005i$. In fig. S3 we show the dependence of the radiation torque, Γ_{rad}, and the transverse trap stiffness, k_x, as a function of the particle radius. As expected, as soon as the chiral parameter has an imaginary part an optical torque is transferred to the particle even for linearly polarized incident light. The transverse stiffness follows a behaviour similar to the non-absorbing case, but with different quantitative values due to light absorption in the trap.

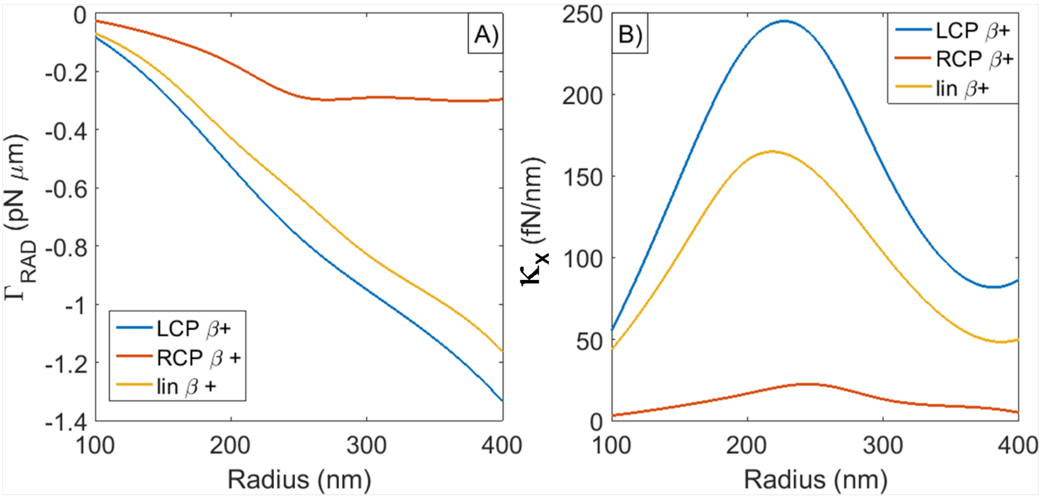

Figure S3: Optical torque (a) and transverse trapping stiffness (b) for a nanosphere with both real and imaginary part of $\beta = 0.05 - 0.005i$. The trapping is achieved with a laser power of 10 mW, $\lambda = 632$ nm, and a TEM_{00} Gaussian profile, focussed by a lens with NA = 1.2.

References

[1] Bohren, C. F. Light scattering by an optically active sphere. *Chemical Physics Letters* **29**, 458–462 (1974).

[2] Bohren, C. F. & Huffman, D. R. *Absorption and scattering of light by small particles* (John Wiley & Sons, 1998).

[3] Luebbers, R., Langdon, H. S., Hunsberger, F., Bohren, C. F. & Yoshikawa, S. Calculation and measurement of the effective chirality parameter of a composite chiral material over a wide frequency band. *IEEE transactions on antennas and propagation* **43**, 123–130 (1995).

[4] Ro, R., Varadan, V. & Varadan, V. Electromagnetic activity and absorption in microwave chiral composites. In *IEE Proceedings H (Microwaves, Antennas and Propagation)*, vol. 139, 441–448 (IET, 1992).

[5] Jones, P. H., Maragò, O. M. & Volpe, G. *Optical tweezers: Principles and applications* (Cambridge University Press, Cambridge, 2015).

[6] Borghese, F., Denti, P., Saija, R. & Iatì, M. A. Optical trapping of nonspherical particles in the T-matrix formalism. *Opt. Express* **15**, 11984–11998 (2007).

[7] Saija, R., Iatí, M. A., Giusto, A., Denti, P. & Borghese, F. Transverse components of the radiation force on nonspherical particles in the T-matrix formalism. *Journal of Quantitative Spectroscopy and Radiative Transfer* **94**, 163 – 179 (2005).

[8] Pfeifer, R. N., Nieminen, T. A., Heckenberg, N. R. & Rubinsztein-Dunlop, H. Colloquium: Momentum of an electromagnetic wave in dielectric media. *Reviews of Modern Physics* **79**, 1197 (2007).

[9] Neves, A. A. R. *et al.* Electromagnetic forces for an arbitrary optical trapping of a spherical dielectric. *Optics Express* **14**, 13101–13106 (2006).

[10] Richards, B. & Wolf, E. Electromagnetic diffraction in optical systems. ii. structure of the image field in an aplanatic system. *Proc. R. Soc. A: Mathematical, Physical and Engineering Sciences* **253**, 358–379 (1959).

[11] Borghese, F., Denti, P., Saija, R. & Iatì, M. A. Radiation torque on nonspherical particles in the transition matrix formalism. *Optics Express* **14**, 9508–9521 (2006).
